# Cough hypersensitivity as a neuro-immune interaction

**DOI:** 10.1186/s13601-015-0069-4

**Published:** 2015-07-15

**Authors:** Woo-Jung Song, Yoon-Seok Chang

**Affiliations:** Department of Internal Medicine, Seoul National University College of Medicine, 101 Daehak-ro, Chongno-gu, Seoul, 110-744 South Korea; Institute of Allergy and Clinical Immunology, Seoul National University Medical Research Center, Seoul, South Korea; Department of Internal Medicine, Seoul National University Bundang Hospital, Seongnam, Gyeonggi-do South Korea

**Keywords:** Cough, Hypersensitivity, Immune, Neuron, Interaction

## Abstract

Cough is an intrinsic protective reflex. However, chronic cough affects a considerable proportion of general population and has a major impact on quality of life. A recent paradigm shift to ‘cough hypersensitivity syndrome’ suggests that chronic cough arises from hypersensitivity of the airway sensory nerves. As cough reflex is determined by interaction of the nervous system with immune system, persistent dysregulation of one or both of these systems may lead to chronic cough hypersensitivity. Here we review the current evidence for the neuro-immune interactions that underlie cough hypersensitivity and discuss future therapeutic strategies.

## Introduction

Cough has bi-directional health effects; it is both an essential defence mechanism that protects the airways from harmful inhalation or aspiration [1], and is also one of the most troublesome symptoms for which patients seek medical attention [2]. The epidemiological burden of chronic cough is substantial, affecting approximately 10 % of the general adult population [3]. Furthermore, chronic cough is a significant clinical problem, as it poses significant impairment to quality of life [4, 5] and challenges to clinicians [6]. However, cough treatments remain less than satisfactory [7]; recent internet surveys of 1120 respondents from 29 European countries found that most patients report very limited effectiveness of current cough medication [8].

Cough is also associated with severity in various chronic airway diseases [9]. In subjects with asthma, poor control was associated with concomitant chronic cough [10, 11]. In ECRHS phase I-II follow-up studies, chronic cough/phlegm were strong markers for individuals suffering from moderate/severe asthma [12]. These findings warrant further understanding of cough pathophysiology and its roles in other airway diseases.

Based on current anatomical diagnostic protocols, clinical practice for chronic cough has been successful [13, 14]. However, it has also been realized that a substantial proportion of chronic cough patients (12-42 %) have cough without identifiable cause, termed idiopathic or refractory cough [15]. This gap indicates the necessity for paradigm change. We may need to further elucidate the mechanism of ‘cough’, as refractoriness may originate from dysregulation in the cough reflex itself. In this regard, a new term, ‘cough hypersensitivity syndrome’, has been proposed to suggest that chronic cough arises from hypersensitivity of airway sensory nerves [16–19].

### Intrinsic nature of chronic cough

As cough is an intrinsically protective reflex, chronic cough could be a protective response against persistent harmful tussigen exposure; however, in the absence of harmful exposure, chronic cough is rather a mal-adaptive response.

In clinical observation, chronic cough patients frequently report that cough is provoked by trivial stimuli such as ‘cold air’, ‘singing/talking’ or ‘fatigue/stress’ [20, 21], which is a hypersensitive cough response to non-tussive stimuli (allotussia) [17]. Another type of hypersensitivity is hypertussia, an increase in cough sensitivity in response to a tussigen [17], which is observed in tussigen inhalation challenge tests [22]. The term ‘hypersensitivity’ in cough is not a synonym for hypersensitivity in allergy, which is the alteration in immunologic response to innocuous environmental antigens [23]. However, considering both cough reflex and immune response have intrinsically protective roles, it is not surprising that chronic cough and allergies frequently overlap, such as in eosinophilic bronchitis, asthma or rhinitis.

**Fig. 1:**
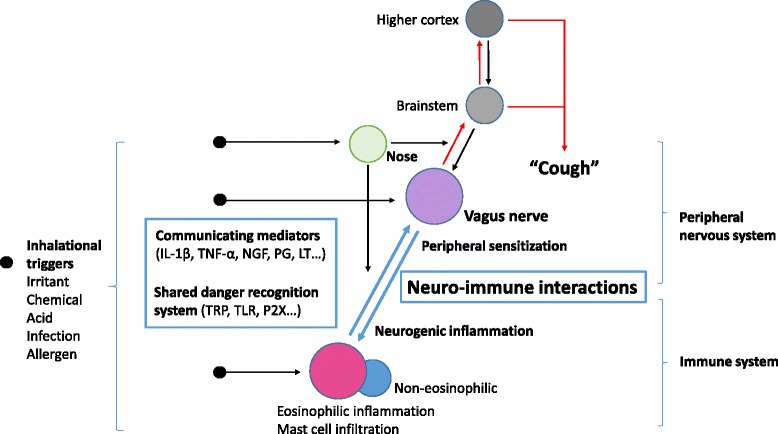
Cough hypersensitivity as a neuro-immune interaction. Schematic presentation of interrelationships between major components in cough reflex pathway, particularly in relation to neuro-immune interaction (marked as bold fonts, closed circles, box, and blue lines). Inhalational triggers may stimulate each of peripheral nervous and immune systems. Activated vagal sensory neurons may induce subsequent immune activation (neurogenic inflammation). Also activated immune systems lead to the up-regulation of cough responses (peripheral sensitization). Further interactions are mediated by communicating mediators and shared danger recognition systems between two systems. Nasal afferents may play modulatory roles in cough hypersensitivity. Modified with permission from Asia Pac Allergy 2014;4:3–13 [19]

Cough reflex is primarily a neuronal response but regulated by interaction with immune system, as both the neuronal and immune systems coordinate to protect the host from exogenous dangers [24]. We suppose that chronic cough hypersensitivity results from persistent dysregulation of either or both systems (Fig. 1). Here we briefly review current evidence for and possible neuro-immune interactions underlying cough hypersensitivity, as well as future therapeutic strategies.

## Review

### Pathologic evidence for cough hypersensitivity in chronic cough

The study by Boulet and colleagues (1994) was the first to investigate the airway pathology of patients suffering from chronic cough [25]. They aimed to compare the degree of airway inflammation in bronchial biopsy tissues and bronchoalveolar lavage fluid (BALF) between non-asthmatic chronic cough patients and healthy controls. Relative to controls, samples from patients with cough had greater numbers of inflammatory cells (particularly mononuclear cells), and displayed epithelial desquamation, submucosal fibrosis, swelling of mitochondria, dilatation of smooth endoplasmic reticulum, and increased nuclear metabolic activity. However, there was no significant difference according to cause of chronic cough (postnasal drip [PND] syndrome or gastroesophageal reflux [GER]). In their BALF, mast cells were more frequent in non-asthmatic cough patients than in controls [25]. Later studies by Niimi and his colleagues also found that mast cell hyperplasia was a distinctive feature in non-asthmatic chronic cough patients [26].

The first study on airway neuronal pathology was reported by O’Connell and colleagues in 1995 [27]. They examined 16 patients with idiopathic persistent cough and eight healthy controls, and found significantly higher calcitonin-gene-related peptide (CGRP)-containing nerve density in idiopathic cough patients. In a further study of 29 chronic cough patients and 16 controls, the expression of transient receptor potential vanilloid-1 (TRPV1), a well-known cough receptor, was increased in the bronchial epithelial nerves of chronic cough patients compared to controls [28]; interestingly, there was no clear difference in pathologic profiles among various etiologic subgroups and/or idiopathic cough. Total nerve density, defined by PGP 9.5 immunostaining, did not significantly differ between cough patients and controls, in both studies [27, 28].

Induced sputum and BALF analyses were also performed by several groups. Notably, there was considerable similarity in the cellular and biochemical profiles among various etiologic subgroups of chronic cough. Jatakanon et al. found increased TNF-α and IL-8 levels in induced sputum in both idiopathic cough and non-asthmatic cough patients [29]. In BALF, McGarvey and colleagues observed an increase in eosinophils, mast cells and histamine levels among non-asthmatic chronic cough patients compared to healthy controls [30]. In *ex vivo* studies using BALF cells, mast cells obtained from chronic cough patients were more responsive to CGRP stimulation, irrespective of their aetiology (asthmatic or non-asthmatic cough) [31]. In studies by Chaudhuri et al., PGE2, LTB4, and cys-LT were expressed at greater levels in patients with cough of any cause [32]. Birring et al. also found high PGE2 and PGD2 levels in all categories of chronic cough [33].

From this review we are unable to conclude that different aetiologies of chronic cough have identical pathologic profiles, due to relatively small sample sizes and different methodologies among studies. However, a considerable similarity in cellular and biochemical profiles suggests a common pathophysiologic process. The evidence indicates that neuronal activation occurs frequently within the airways of chronic cough patients, demonstrated by common findings of mast cell infiltration and increased CGRP, TRPV1, and prostaglandins. Mast cells are innate immune cells that form a functional unit with sensory nerves for tissue surveillance including airways [34, 35]. CGRP is a neuropeptide generated from neurogenic inflammation of sensory nerves, and BALF CGRP levels significantly correlate with capsaicin cough sensitivity [36]. PGE2 and PGD2 are cough reflex sensitizers and can also act as tussigens [37, 38].

### Immune systems in cough hypersensitivity

Dysregulation of the immune system may lead to cough hypersensitivity, as in the well-known example of eosinophilic airway inflammation.

Eosinophilic bronchitis has been identified as a frequent cause of chronic cough, even in the absence of asthma [39]. A causal relationship is supported by a long clinical experience with corticosteroid therapy in these patients. In clinical studies, changes in sputum eosinophilia following inhaled corticoid therapy significantly correlate with changes in capsaicin cough sensitivity [40]. The contribution of eosinophils is also supported by experimental findings, as these cells produce eosinophil granule proteins and inflammatory mediators like PGE2, cys-LT or substance P, which lead to cough reflex sensitization. Eosinophil-derived granule proteins directly stimulate vagal pulmonary C-fibres [41], and major basic proteins (MBP) elicit the release of substance P from cultured dorsal root ganglion neurons [42]. In addition, MBP can activate human lung mast cells via a non-IgE-dependent pathway, leading to the release of histamine and PGD2 [43]. In turn, the release of neuropeptides such as substance P and CGRP leads to the chemotaxis of eosinophils [44]. In guinea pig models, eosinophils are co-localized with airway nerves after allergen challenge [45].

Meanwhile, evidence indicates that eosinophils are not a pre-requisite for cough hypersensitivity, at least in asthma. In anti-IL-5 antibody trials for refractory eosinophilic asthma, mepolizumab treatment suppressed sputum eosinophilia and reduced severe asthma exacerbations, but failed to improve cough severity compared to placebo [46]. This finding directly contrasts the effects of systemic corticosteroid therapy (prednisolone 30 mg daily for two weeks), which significantly improved inflammatory markers and cough scores in refractory eosinophilic asthma patients. These results lead to the speculation that immune cells other than eosinophils, particularly mast cells, contribute to cough in asthma patients [47]; this idea is supported by previous reports of increased mast cell numbers in chronic cough [25, 26, 30]. These findings also warrant further investigation of whether anti-IL-5 (eosinophil-specific reduction therapy) is effective in non-asthmatic eosinophilic bronchitis.

Few studies have examined the pathogenesis of non-asthmatic eosinophilic bronchitis. This condition is less frequently accompanied by IgE sensitization to inhalant allergens (atopy) than eosinophilic asthma [47]. It is also unlikely to originate from nasal eosinophilic inflammation, as sputum eosinophilia did not frequently accompany nasal eosinophilia and responded well to inhaled corticosteroid therapy [40]. Potential relationships between airway eosinophilia and reflux diseases have been reported [30, 48], but warrant further clarification. In pathologic studies, degrees of submucosal eosinophil and mast cell infiltration were similar between non-asthmatic eosinophilic bronchitis and asthma, but eosinophilic bronchitis involved much less mast cell infiltration in airway smooth muscle [49]. This difference from asthma highlights need to elucidate the pathogenesis of non-asthmatic eosinophilic bronchitis. In addition, the potential role of mast cells [25, 26, 30, 31] also warrants further investigation in this condition.

Inflammatory mediators such as IL-1β, TNF-α and nerve growth factor (NGF) released from immune cells can directly sensitize sensory neurons [50–52], and thus could lead to hypersensitivity in the cough reflex. However, whether and how non-eosinophilic inflammation contributes to neuronal sensitization remains unclear.

### Peripheral nervous system in cough hypersensitivity

The cough reflex is mediated by peripheral sensory nerves, mostly within the extrapulmonary airways (larynx, trachea and large bronchus). Thus, repeated stimulation or dysregulation of sensory neurons could lead to cough hypersensitivity. Here we briefly review the mechanisms of peripheral cough reflex pathway.

The various sensory nerves involved in the cough reflex originate from the vagal nodose and jugular ganglia. The sensory fibres terminate within the airway epithelial layers, and recognize incoming harmful signals. Activation triggers an action potential, which is relayed along afferent pathways to the nucleus tractus solitarius (nTS) in the convergence centre. Afferent signals are summed, and efferent signals for the act of coughing are then decided [53].

There are two subtypes of vagal afferents, depending on how they respond to different stimuli [54]. The sensation of mechanical stimuli is mainly mediated by a low-threshold mechanoreceptor, also responsive to low pH through acid-sensing ion channels, but usually not to chemical irritants like capsaicin [55, 56]. This mechanoreceptor is fast-conducting and does not produce neuropeptides under normal conditions. Stimulation of mechanoreceptors induces the cough reflex regardless of general anaesthesia [57], and thus they are thought to mediate intrinsic protective roles for the lower airways against acid or foreign body aspiration.

The sensation of chemical irritants and endogenous inflammatory mediators is mostly mediated by bronchial C-fibres [54]. C-fibres play a chemosensitive function by expressing various receptors or channels, such as TRPV1 or TRP ankyrin-1 (TRPA1). TRPV1 is the most well-known receptor for cough, which responds to high temperature, low pH and capsaicin [58]. TRPA1 responds to cold temperature and a variety of irritants including cigarette smoke or acrolein [59]. C-fibre tussigenic function is up-regulated (sensitized) by inflammatory mediators, and appears to be maintained only during consciousness [55]. Thus, C-fibres are understood to mediate adaptive cough responses in pathologic conditions, making them the likely neuronal basis of cough hypersensitivity and thus appropriate therapeutic targets at peripheral levels. Pathologic changes at higher levels of nervous system, such as brainstem or brain cortex, are also supposed to augment cough hypersensitivity significantly [17]; however, this topic will not be discussed here.

Acute stimulation of sensory neurons leads to local activation of immune cells and also up-regulation of cough receptors at the peripheral level (peripheral sensitization). However, it is unclear whether repeated stimulation of sensory neurons is sufficient to cause persistent neuropathic changes in human cough afferent pathways (chronic cough hypersensitivity). In a primate model of allergic asthma, sensitization and repeated exposure to house dust mites induced intrinsic increases in neuronal excitability in nTS [60]. In young guinea pigs, repeated second-hand tobacco smoke exposure increased excitability of the second order neurons in the nTS via the production of substance P [61].

Respiratory infection is another candidate for developing cough hypersensitivity. Acute infection with human rhinovirus in d-IMR-32 neuronal cell lines up-regulated expression of cough receptors including TRPV1 and TRPA1 [62]. During H1N1 infection, plasma NGF levels correlated with the duration of cough [63]. In an autopsy study of mycoplasmal panencephalitis accompanied by fever and cough, *Mycoplasma pneumoniae* was found to have infected microglia, oligodendrocytes and neurons [64]. However, whether respiratory infection leads to neuropathic changes and chronic cough hypersensitivity remains undetermined.

Nutritional factors could also be involved in cough hypersensitivity, by mediating sensory neuropathy. Unexplained chronic cough patients with vitamin B-12 deficiency had more hyperresponsiveness to histamine and higher NGF immune-reactive score in oropharyngeal biopsy, compared to those without vitamin B-12 deficiency [65]. Also cough visual analogue scale and histamine hyperresponsiveness were significantly improved by 2-month supplementation with vitamin B-12, particularly among those with the deficiency [65]. Potential roles of iron deficiency were also suggested in female patients with unexplained chronic cough [66].

Despite the fundamental roles of neuronal circuits in cough reflex regulation, evidence from human studies is lacking. While their function is clear from cough challenge studies [22], the pathology of airway sensory nerves in chronic cough is under-studied. As discussed earlier, CGRP and TRPV1 expression in airway nerves correlate with cough severity and duration [27, 28], but these biopsy samples were mostly taken from carina and large bronchi, not laryngeal mucosa, which are closer to the intrinsic function of the cough reflex and have a high density of sensory nerve fibres [67]. Moreover, to our knowledge, there are no reports of changes in the nervous tissues at the ganglionic or brainstem levels in relation to cough sensitivity. Given the recent identification of novel cough receptors [68], further studies are encouraged in humans.

### Neuro-immune interactions in cough hypersensitivity

The immune and nervous systems have distinct roles, but closely interact with each other to protect the host, including through the cough reflex. As discussed previously, dysregulation in either or both systems may lead to cough hypersensitivity. Eosinophilic or Th2 inflammation may directly sensitize nerves, by releasing eosinophil granule proteins, PGE2, cys-LT or neuropeptides. Infiltration of mast cells could be a cause or sign of sensory hypersensitivity in the airways. Thus, ongoing immunologic hypersensitivity would lead to persistent sensitization of sensory neurons.

Conversely, neurogenic inflammation initiated by primary stimulation of afferent nerve endings may also in turn locally activate the immune system by releasing neuropeptides like CGRP and substance P, which can induce vasodilation and promote oedema [69, 70]. They can also attract and activate immune cells including eosinophils, mast cells, dendritic cells or T cells [44, 71–73]. Increased CGRP could bias Langerhans cell functions toward Th2-type immunity in skin inflammation [74], although this effect remains to be examined in the airways.

Another important interaction between the two systems is a shared danger recognition system. Toll-like receptors (TLRs), well-known as detectors of microbial components in innate immune cells, are also expressed in nociceptive neurons. In particular, TLRs 3, 4, 7 and 9 expression and function in neuronal cells have recently been demonstrated [75–78]. Stimulation of these TLRs in sensory neurons mediates pain, itch, or sensitization to other kinds of stimuli. At the same time, TLR stimulation in innate immune cells leads to inflammatory cascades, resulting in synergistic protection.

TRP channels, which mediate neurogenic inflammation in sensory neurons, have recently been identified as being expressed and functional in non-neuronal cells such as airway epithelium, smooth muscle cells, or lung fibroblasts [79, 80]. TRPA1, which mediates the cough response in humans [59], is also expressed in non-neuronal cells and mediates non-neurogenic inflammation in the airways [79]. Increased TRPV1 expression in bronchial epithelium correlates with the severity of asthma, and TRPV1 agonist stimulation in bronchial epithelium induces IL-8 release in a dose-dependent manner [80].

ATP and corresponding purinergic receptors are another shared danger and recognition mechanism. ATP is a danger signal generated during cell injury, and can be recognized by both immune and neuronal cells via purinergic receptors like P2X. In the immune system, extracellular ATP stimulation of P2X7 receptors induces mast cell activation [81], IL-1β release in macrophages [82], and the proliferation of B and T cells [83, 84]. Sensory neurons can also recognize extracellular ATP via P2X3 receptors, and mediate cough responses to tussigens in guinea pigs [85, 86]. Importantly, the P2X3 receptor antagonist AF-219 significantly reduced the frequency of cough in a very recent phase II trial in refractory chronic cough patients [87].

However, how these interactions are involved in cough hypersensitivity remains unclear. Moreover, whether blockade of communicating mediators (TNF-α, IL-1β, or NGF) or shared danger recognition receptors (TLRs, TRPs, or P2Xs) as an effective strategy for resolving cough hypersensitivity also deserves further investigation.

### Nasal determinants of the cough reflex

We here discuss upper airway cough syndrome as a separate part, as this entity is supposed to have a distinct type of interaction. Upper airway cough syndrome is regarded as a frequent cause of chronic cough, but the pathophysiology remains to be fully elucidated [88]. In the past, cough and comorbid rhinitis was attributed to PND to the pharyngolaryngeal region, directly stimulating the cough response. However, PND is a common physiologic phenomenon, and only a minority of patients with purulent rhinosinusitis complain of cough [89]. Thus, PND syndrome was later renamed upper airway cough syndrome, reflecting its complex mechanisms and highlighting the role of nasal determinants in cough regulation.

Nasal mucosa express various TLRs and cough receptors such as TRPV1, TRPA1 and melastatin-8 (TRPM8), and thus sense various kinds of stimuli. However, direct stimulation of the nasal afferent does not induce cough, but only the sneeze reflex [88]. Rather, nasal afferent stimulation modulates cough reflex indirectly; in inhalational tussigen challenges, the cough reflex becomes sensitized by prior intranasal histamine or capsaicin stimulation [90]. Similarly, in allergic rhinitis patients, the cough reflex is sensitized during the pollen season [91]. In this regard, we speculate that up-regulation of the cough reflex during nasal afferent stimulation minimizes the spread of harmful stimuli from the nasal cavity to the lower airways. Repeated nasal trigeminal stimulation by capsaicin also induces *c-fos* expression in the nTS, indicating the potential contribution of upper airway neurogenic inflammation in central sensitization of cough [92]. More interestingly, the nasal challenge with menthol, a TRPM8 agonist, ‘desensitizes’ the cough reflex [93]. Collectively, these findings provide evidence that the nasal trigeminal afferent is involved in cough regulatory mechanisms, which were previously thought to be mediated exclusively by vagal afferent nerves. In turn, these findings suggest nasal modulation of the cough reflex has a distinct role in cough hypersensitivity.

### Clinical appraisal: current and future therapeutic strategies

Based on the concept of cough hypersensitivity and neuro-immune interaction, here we review current and future therapeutic strategies for cough. Considering its bi-directional health effects, the goal of therapy would be normalization of hypersensitivity (pathologic cough) rather than overall suppression of cough pathways.

To date, most anti-tussive agents are centrally acting and non-selective; some of the most effective anti-tussive medications are opiates [94]. In a four-week randomized double-blind placebo-controlled trial, slow-release morphine sulphate (5 mg twice daily) rapidly and significantly reduced daily cough scores [95]. However, the mechanism of action is not clear, but unlikely due to sedation [96]. They often have undesirable side effects, and their effectiveness varies among individuals.

Gabapentin has recently been highlighted as having a therapeutic benefit in chronic refractory cough [97]. In a ten-week randomized double-blind placebo-controlled trial, gabapentin (maximum tolerable daily dose of 1800 mg) significantly improved cough-specific quality of life. However, gabapentin had a high rate of side effects (31 %). Another limitation of opiates or gabapentin is that they do not suppress peripheral cough sensitivity to citric acid or capsaicin [95, 97], indicating that they may not suppress cough in cases of unresolved peripheral triggers or inflammation.

Dextromethorphan is another centrally-acting medication used for a long time, which exerts anti-tussive effects by the structural component of codeine and also the N-methyl D aspartate receptor antagonist function. It showed some efficacy in clinical trials [94], attenuated capsaicin cough response [98], but has safety concerns [99].

Thus, selective blockade of peripheral cough receptors and pathways is expected to be the next breakthrough. However, a TRPV1 receptor antagonist (SB-705498) did not reduce objective cough frequency, despite reducing capsaicin cough reflex sensitivity [100]. These findings raise the question of whether specific cough receptor blockade is an appropriate strategy. However, P2X3 receptor antagonist (AF-219) yielded very promising results [87], although its efficacy in blocking the peripheral cough circuit has not yet been examined. Recent increase in the number of clinical trials for novel therapeutics is encouraging.

Considering diverse implication of cys-LTs in airway inflammation [101], therapeutic effects of leukotriene receptor antagonist (LTRA) may be considered. LTRAs such as montelukast or zafirlukast have shown significant clinical efficacy in improving cough and/or capsaicin cough sensitivity among patients with cough variant asthma or non-asthmatic eosinophilic bronchitis [102–105]. However, roles of LTRA as non-specific anti-tussive agents have been inconclusive, or is unlikely at present [104, 106, 107]. In a recent large-scale randomized trial on 276 patients with post-infectious cough, montelukast did not show any significant difference in improving cough outcomes, compared to placebo [108].

Non-pharmacological intervention is suggested as a safe and effective option in normalizing cough hypersensitivity, although further validation is required [109]. In a randomized placebo-controlled trial on 87 refractory cough patients, speech pathology intervention for 2 months significantly improved cough scores, compared to placebo intervention (general health lifestyle advice) [110]; the positive effects were also shown in later studies, including further benefits in improving cough sensitivity [109, 111]. Nutritional intervention and weight reduction may also have beneficial roles in susceptible patients [65, 66, 112].

**Fig. 2:**
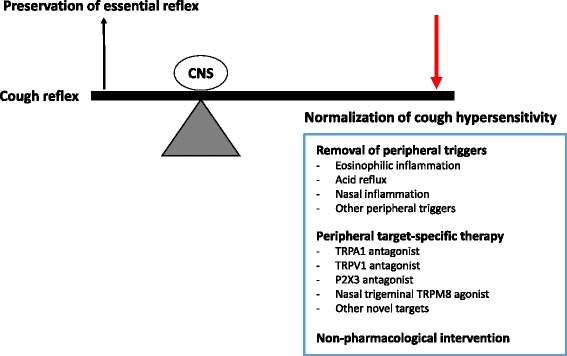
Clinical approach from the concept of cough hypersensitivity. Abbreviations: CNS, central nervous system; TRPA1, transient receptor potential ankyrin-1; TRPV1, transient receptor potential vanilloid-1; TRPM8, transient receptor potential melastatin-8

At present, the best strategy would be the combination of 1) identification and treatment of peripheral triggers (eosinophilic inflammation, acid reflux, or nasal inflammation), 2) appropriate anti-tussive medication, and 3) non-pharmacological intervention (Fig. 2). However, current anti-tussives may not down-regulate the ‘hypersensitivity’ of the pathologic cough reflex, but suppress overall cough pathways at central levels. We expect ongoing research and trials to finally bring a new strategy for chronic cough patients.

## Conclusions

Anatomic diagnostic protocol was the first breakthrough in practice of chronic cough. A recent paradigm shift into ‘cough hypersensitivity’ as an intrinsic mechanism for chronic cough provides new opportunities to discover the next breakthrough. As reviewed here, the nervous system is fundamental in regulating the cough reflex, and activation of sensory neurons can lead to acute immune activation, and if repeated, may lead to a chronic neuronal hypersensitive state. In turn, activation of the immune system can strongly sensitize the nervous system leading to cough hypersensitivity; roles of eosinophils and mast cells have been suggested. Further potential interactions between the two systems may reside in shared danger recognition systems. We expect further elucidation of neuro-immune interactions to lead to new therapeutic strategies for chronic cough.
